# Nutritional composition and prediction of energy values of poultry offal meal and meat and bone meal for broilers

**DOI:** 10.1007/s11250-025-04580-8

**Published:** 2025-08-01

**Authors:** Camilla Roana Costa de Oliveira, Carlos Bôa-Viagem Rabello, Paulo Borges Rodrigues, Claudia da Costa Lopes, Bruno Araújo Silva, Elainy Cristina Lopes, Maria do Carmo Mohaupt Marques Ludke, Lilian Francisco Arantes de Souza, Júlio Cézar dos Santos Nascimento, Webert Aurino da Silva, Apolonio Gomes Ribeiro, Lucas Rannier Ribeiro Antonino Carvalho, Marcos José Batista dos Santos

**Affiliations:** 1 Animal Science Department, Federal Rural University of Pernambuco, Recife, PE Brazil; 2 Animal Science Department, Federal University of Lavras, Lavras, MG Brazil; 3 Animal Science Departament, Federal University of Rio Grande do Norte, Natal, RN Brazil; 4 Animal Science Departament, Federal University of Paraiba, Areia, PB Brazil; 5 Department of Physiology and Pharmacology, Karolinska Institutet - Stockholm Sweden Biomedicum, 5B, Solnavägen 9, Stockholm, S-171 77 Sweden

**Keywords:** Metabolizable energy, Prediction equation, Poultry offal meal, Meat and bone meal

## Abstract

This study evaluated the apparent metabolizable energy (AME) and nitrogen-corrected AME (AMEn) of poultry offal meal (POM) and meat and bone meal (MBM) in broilers at different ages. Three metabolism trials were conducted in the pre-initial (1–8 days), starter (14–22 days), and grower (28–36 days) phases using 840 Cobb-500 broilers. Birds were housed in metabolic cages, and excreta were collected using the partial collection method. The experiment followed a completely randomized design with seven treatments and six replicates, with 10, 6, and 4 birds per phase. Treatments included a control diet and six test diets (80% control + 20% test ingredient: POM 1, 2, 3; MBM 1, 2, 3). AMEn values ranged from 3,189.7 to 3,342.9 kcal/kg in POM and 2,292.1 to 2,345.4 kcal/kg in MBM. Multiple regression equations were developed to predict AMEn. For POM, the best equation was AMEn = 6802 ‒ 213.3 (ether extract) ‒ 127.3 (calcium) + 10.47 (age) (R² = 0.92). For MBM, the equation was AMEn = 2789 ‒ 72 (ether extract) + 14 (calcium) + 20.28 (age) (R² = 0.91). These equations effectively estimate AMEn based on chemical composition and broiler age.

## Introduction

To increase the economic viability of production systems, nutritionists seek alternative feedstuffs that meet the requirements of animals in their different production stages and reduce feed costs (Babatunde et al. 2021). Slaughterhouse by-products like poultry offal meal and meat and bone meal (MBM) are alternative dietary sources of protein and phosphorus (P) that can partially or fully substitute costly ingredients such as soybean meal and dicalcium phosphate (Munoz et al. 2020). In addition to its economic importance, this replacement also addresses environmental concerns, as it represents a proper destination for slaughterhouse waste (Limeneh et al. 2022).

The basic production process of animal-derived meals involves non-edible harvest waste from slaughter, which should be free of foreign materials and pathogenic microorganisms (Leiva et al. 2018).

The production of offal meal involves thermal, chemical, or enzymatic hydrolysis to break down feather keratin and improve digestibility (Mokrejs et al. 2010; Staroń et al. 2017). Viscera is processed through rendering, separating fats from proteins, resulting in a nutrient-rich meal (Zhang et al. 2023;). Autoclaving and alkaline-enzymatic hydrolysis optimize protein degradation and reduce waste (Mokrejš et al. 2010; Dieckmann et al. 2020).

Adequate feed formulation requires information about the chemical composition and metabolizable energy (ME) values at different birds’ ages (Adeola et al. 2018). However, animal-derived meals have a highly variable chemical composition, which depends on the processing, type, and proportions of its components (Silva et al. 2020).

For this reason, chemical composition values presented by Rostagno et al. (2024) and NRC (1994) are not always stable and standardized, and this leads to formulations that might over- or underestimate the diet energy value, ultimately compromising the performance of poultry. Nevertheless, determining the ME of ingredients through in vivo trials is often unfeasible, as this method is costly and requires space and labor. Metabolizable energy (ME) values for feedstuffs can be estimated practically and economically by using prediction equations based on chemical composition. These methods allow for rapid evaluation of energy values while being cost-effective, provided that the equations are established from standardized in vivo measurements (Noblet et al. 2022).

Due to high variability in the chemical composition of animal-derived meals from different raw materials and processing methods (Silva et al. 2020; Najafabadi et al. 2007), three batches of each ingredient (POM and MBM) were evaluated. This approach aimed to capture natural variability among sources and enhance the robustness of the prediction models across production conditions. Since broiler nutrient requirements and digestive efficiency change with age (Adeola et al. 2018; Schneiders et al. 2021), evaluating three distinct growth phases was essential. This enabled more accurate AMEn prediction equations that consider birds’ ingredient composition and age-related digestive capacity.

The present study was developed to determine the chemical composition and energy values of animal-derived meals for broilers at different ages and to estimate AME_n_ values using prediction equations.

## Materials and methods

### Facilities and animal handling

The study was conducted at the Federal Rural University of Pernambuco, specifically in the Laboratory of Non-Ruminant Digestibility, following approval from the ethics committee (process 23082.000496/2015). The experiment had three phases (1–8, 14–22, and 28–36 days), using three poultry offal meals (POM) and three meat and bone meals (MBM) from suppliers in northern and northeastern Brazil. The poultry offal meals originated from broiler chickens, while the meat and bone meals originated exclusively. from bovine sources. Although the suppliers did not disclose precise processing parameters, the meals were produced using standard rendering procedures, which typically involve thermal treatment, fat separation, and grinding, as commonly practiced in the Brazilian rendering industry.

A total of 840 male Cobb 500 chicks were reared on the floor and later transferred to metabolism cages (1.00 × 0.50 × 0.50 m) with controlled temperature, ventilation, and 24 h lighting. Average temperatures were 30.8 °C, 27.5 °C, and 26.8 °C across phases. Feed and water were provided *ad libitum*.

### Experimental design and diets

Broiler chickens were arranged in a completely randomized design with seven treatments and six replicates. The number of birds per experimental unit adjusted for age and space: ten from 1 to 8 days, six from 14 to 22 days, and four from 28 to 36 days. This adjustment is standard in metabolism trials to maintain stocking density, ensure animal welfare, and facilitate accurate excreta collection, optimizing data quality and experimental results efficiency. Treatments in each research phase consisted of a corn and soybean meal-based diet formulated following Rostagno et al. (2011). To meet the nutritional birds’ requirements for each phase (Table 1), six test diets comprised 20% of one of the animal-derived meals plus 80% of the control diet.

**Table 1:** Centesimal and nutritional composition of reference diets

Centesimal composition	Pre-initial	Initial	Growth
Ground corn	50.126	51.262	51.154
Soybean meal	42.000	40.000	39.000
Soy oil	3.725	5.141	6.608
Dicalcium phosphate	1.880	0.403	1.260
Calcitic Limestone	0.850	0.870	0.880
Ground salt	0.400	0.403	0.406
Vitamin and Mineral Supplement^1^	0.400	0.400	-------
Vitamin supplement^2^	---------	---------	0.100
Mineral supplement^3^	---------	---------	0.050
DL Methionine	0.192	0.137	0.273
L-Lysine	0.296	0.191	0.125
L Threonine	0.130	0.075	0.052
Salinomycin Sodium^4^	--------	--------	0.055
Zinc Bacitracin 15%	--------	--------	0.036
Total	100.00	100.00	100.00
Nutritional composition calculated			
AME_n_, kcal/kg	3000	3100	3200
Crude protein, %	22.440	21.770	21.150
Calcium, %	0.920	0.840	0.760
Total Phosphorus, %	0.703	0.630	0.576
Phosphorus Available, %	0.470	0.400	0.350
Sodium, %	0.180	0.180	0.180
Ether Extract, %	6.177	7.582	9.027
Gross Fiber, %	3.789	3.711	3.629
Digestible Amino Acids, %			
Lysine	1.320	1.210	1.130
Methionine	0.650	0.590	0.550
Methionine + Cystine	0.938	0.872	0.826
Threonine	0.860	0.790	0.750
Tryptophan	0.227	0.222	0.216
Arginine	1.025	1.090	1.137
Valine	----	0.722	0.754
Isoleucine	----	0.674	0.704

^1^ Vitamin and mineral supplementation, Composition / kg: Vit A − 13,700 IU; Vit. D − 3,200 IU; Vit. E−35 IU; Vit. Vit. K 4.4 mg; Vit. B1 2.5 mg; Vit. B2 6.5 mg; Vit. B6 5.5 mg; Vit. B12 7 mg; Biotin 96 mg; Folic acid 1.2 mg; Niacia 54 mg; Pantothenic acid 14 mg and Se 300 mg. Mn 90 mg; Zn 80 mg; Fe 30 mg; Cu 10 mg; Iodine 2 mg

_2_Vitamin Supplement (Guarantee levels per kg of product): Vit A 7500000 IU, Vit D3 2500000 IU, Vit E 18000 IU, Vit K3 1200 mg, Thiamine 1500 mg, Riboflavin 5500 mg, Pyridoxine 2000 mg, Vit B12 12500 mcg, Niacin 35 g, Calcium Pantothenate 10 g, Biotin 67 mg. 3Mineral supplement. (Guarantee levels per kg of product). Iron 60 g, Copper 13 g, Manganese 120 g, Zinc 100 g, Iodine 2500 mg, Selenium 500 mg. 4 Monensin Sodium

The AME and AMEn values were determined using the partial excreta collection method, with canvas-lined trays placed under metabolism cages. Birds underwent an adaptation period (four days, except for the 1–8-day phase, which had three), followed by four days of excreta collection. Samples were collected twice daily, stored at ‒20 °C, then pre-dried (55 °C, 72 h), ground, and analyzed. Acid insoluble ash (1%) was used as an indigestibility marker.

Ileal digestibility coefficients were obtained at the end of each phase. Birds were euthanized, and ileal contents were collected from an 18 cm segment, stored at ‒20 °C, pre-dried, ground, and analyzed.

### Chemical analyses

Samples of ingredients, diets, excreta, and ileal digesta were analyzed at the Laboratory of Animal Nutrition in the Department of Animal Science of UFRPE for dry matter, mineral matter, ether extract, and crude fiber contents according to the methodologies described by Silva and Queiroz (2005). While specific numerical procedure codes are not assigned within this reference, the standard chemical analytical techniques for feedstuffs as detailed therein were rigorously followed.

Calcium (Ca) and phosphorus (P) concentrations were determined according to the Official Methods of Analysis of the AOAC (1995), specifically following method 995.11 for Calcium and method 984.27 for Phosphorus.

Gross energy (GE) was obtained using a bomb calorimeter (IKA-C200), following the manufacturer’s operational protocols for calorimetric determination.

Amino acids in the tested ingredients were determined by near-infrared reflectance spectroscopy (NIRS) at Evonik Brasil, using proprietary calibration models based on extensive wet chemistry analyses. NIRS measurements were performed under standard operational conditions, ensuring precision and accuracy consistent with commercial industry practices for feedstuff analysis.

To quantify the acid-insoluble ash (AIA) marker concentration in feeds and excreta, we followed the methodology proposed by Van Keulen and Young (1977).

## Calculations and statistical analysis

AME values were calculated and adjusted for nitrogen balance (AMEn) using the equations of Matterson et al. (1965) and Hill and Anderson (1958). Metabolizability coefficients (MCDM, MCCP, MCGE) and ileal digestibility coefficients (IDCDM, IDCCP, DDM, DCP) were determined according to Sakomura and Rostagno (2007).

The experiment followed a completely randomized design (CRD) with seven treatments and six replicates per treatment in each phase. For the statistical analysis of AME, AMEn, metabolizability coefficients, and ileal digestibility coefficients, the data were further analyzed in a 3 × 6 factorial arrangement (three broiler ages × six animal meal samples), considering age and ingredient source as fixed effects.

Prior to performing ANOVA, data normality and homogeneity of variances were verified using the Shapiro-Wilk and Levene’s tests, respectively. When significant differences were detected, treatment means were compared using Tukey’s test at a 5% significance level.

All statistical procedures were conducted using SAS software (SAS Institute, 2004). Nutrient values were adjusted to 92% dry matter (Dale et al. 1993) and subjected to Pearson’s correlation analysis to identify the variables most strongly associated with AMEn across phases.

Multiple regression analyses were then performed to develop AMEn prediction equations for poultry offal meal (POM) and meat and bone meal (MBM), including an all-phase model incorporating age as an independent variable. Variable selection in the regression models followed a backward elimination procedure, adopting a P-value threshold of 0.05 for retention. Multicollinearity among explanatory variables was assessed using the variance inflation factor (VIF), with VIF values below 10 considered acceptable.

## Results

The chemical composition of POM and MBM are described in Table 2. The maximum DM values observed in POM and MBM were 94.74% and 94.47%, respectively. The CP content in POM varied from 61.08 to 61.99, while in MBM this component ranged from 41.27 to 42.76. Maximum EE contents in POM and MBM were 13.33 and 12.18%, respectively. Considering the six tested chemical composition meals, MM, Ca, and P were the components whose concentrations were most varied.

**Table 2:** Chemical and amino acid composition based on natural matter of poultry offal meal (POM) and meat and bone meal (MBM) used in the experiment

Animal meal						
Variable	POM 1	POM 2	POM 3	MBM 1	MBM 2	MBM 3
Dry matter, %	93.81	92.39	94.74	94.01	92.45	94.47
Crude protein, %	61.99	61.25	61.08	42.76	41.27	41.68
Ether Extract, %	12.55	13.33	12.47	11.17	12.18	11.31
Mineral Matter, %	12.08	10.87	11.59	38.99	41.23	43.77
Gross Fiber, %	3.25	2.96	2.55	2.35	2.84	2.67
Gross Energy, kcal/kg	4863	4855	4828	3391	3342	3234
Calcium, %	3.23	2.7	4.57	11.95	13.17	13.02
Phosphorus, %	2.22	1.82	2.68	6.45	7.0	7.48
Digestible Amino Acids, %						
Lysine	3.550	3.187	3.226	2.021	1.747	1.347
Methionine	1.203	1.132	1.067	0.522	0.456	0.380
Met + Cist	1.776	1.724	1.771	0.799	0.662	0.574
Threonine	2.288	2.102	2.129	1.199	1.038	0.980
Tryptophan	0.524	0.483	0.519	0.185	0.134	0.106
Arginine	4.219	3.953	3.909	3.234	3.017	2.703
Valine	2.734	2.590	2.561	1.612	1.349	1.234
Isoleucine	2.264	2.121	2.057	1.010	0.844	0.745
Leucine	4.168	3.796	3.796	2.237	1.911	1.837
Histidine	1.195	1.030	1.083	0.624	0.503	0.450
Phenylalanine	2.352	2.180	2.197	1.287	1.106	1.042
Cystine	0.649	0.553	0.715	0.285	0.209	0.190

The AME and AME_n_ values were significant for the *meal* and *age* factors (Table 3), with higher means found in POM, where AME_n_ ranged from 3,189.7 to 3,342.9 kcal/kg. MBM had AME_n_ contents ranging from 2,292.1 to 2,345.4 kcal/kg. Despite the lack of interactions, the MC_DM_ was significant (*P* < 0.01) for both factors (*age* and *meal*) which increased with birds’ age, with higher values found for POM. CP coefficients also increased as the birds’ age, with higher values found for MBM. The MC_GE_, in turn, showed an interaction between the studied factors (*P* = 0.04), but the average of treatments did not show significant differences.

**Table 3:** Mean values of metabolizable energy (AME) and (AMEn), coefficients of dry matter metabolizability (MCDM), crude protein (MCCP) and gross energy (MCGE) of animal meal at three phases, expressed in natural matter

	Animal meal						*P*-Value				
AME, kcal/kg											
Age	POM 1	POM 2	POM 3	MBM1	MBM2	MBM3	Mean	SEM	Age	Meal	Age x Meal
1–8	3390	3297	3381	2168	2083	2072	2732 ^C^	23.247	0.001	0.001	0.914
14–22	3561	3412	3462	2264	2221	2267	2865 ^B^				
28–36	3760	3624	3518	2603	2571	2682	3126 ^A^				
Means	3570 ^a^	3444 ^a^	3454 ^a^	2345 ^b^	2292 ^b^	2340 ^b^					
AME_n_, kcal/kg											
1–8	3123	3094	3147	2019	1825	1811	2503 ^C^	24.234	0.001	0.001	0.513
14–22	3373	3239	3174	2136	2125	2231	2713 ^B^				
28–36	3530	3396	3247	2352	2391	2479	2899 ^A^				
Means	3342^a^	3243^a^	3189^a^	2169^b^	2113 ^b^	2174 ^b^					
MC_DM_, %											
1–8	61.05	58.14	58.06	42.96	38.17	43.06	50.24 ^C^	1.295	0.001	0.003	0.701
14–22	63.67	63.53	62.45	45.67	46.53	45.50	54.55 ^B^				
28–36	76.04	72.08	76.25	63.24	66.00	63.61	69.54 ^A^				
Means	66.92 ^a^	64.58 ^a^	65.59 ^a^	50.63 ^b^	50.24 ^b^	50.72 ^b^					
MC_CP_, %											
1–8	49.92	49.91	49.93	49.96	50.29	50.37	50.06 ^C^	0.03	0.001	0.001	0.611
14–22	55.95	55.92	56.08	56.17	56.11	56.38	56.10 ^B^				
28–36	59.52	59.45	59.66	59.58	59.56	59.57	59.55 ^A^				
Means	55.13 ^c^	55.10 ^c^	55.22 ^bc^	55.23 ^bc^	55.32 ^ab^	55.44 ^a^					
MC_GE_, %											
1–8	64.2 ^abA^	63.7 ^abA^	65.1 ^aA^	59.5 ^abA^	54.6 ^bB^	56.1 ^abB^	60.55 ^C^	0.973	0.001	0.084	0.044
14–22	69.3 ^aA^	66.7 ^aA^	65.7 ^aA^	62.9 ^aAB^	63.6 ^aA^	68.9 ^aA^	66.24 ^B^				
28–36	72.6 ^aA^	69.9 ^aA^	67.2 ^aA^	69.3 ^aA^	71.5 ^aA^	76.6 ^aA^	71.23 ^A^				
Means	68.74 ^a^	66.80 ^a^	66.06 ^a^	63.97 ^a^	63.25 ^a^	67.22 ^a^					

Different lowercase letters in the row and uppercase letters in the column. differ by the Tukey test at 5% probability; POM−poultry offal meal

MBM− meat and bone meal; P−value: probability; CV: coefficient of variation; IxF: probability of age factor interaction and flour factor

The IDC_DM_ (*P* < 0.01) and IDC_CP_ (*P* < 0.01) were significant for the meal and age factors, but there was no interaction between them (Table 4). The former variable increased from the pre-starter to the starter phase, stabilizing in the final phase, while IDC_CP_ was lowest in the last life stage. Treatments with POM showed a higher IDC_DM_, whereas IDC_CP_ was higher in the MBM-containing treatments.

**Table 4:** Coefficients of ileal dry matter digestibility (IDC_DM_), crude protein (IDC_CP_), digestible dry matter (D_DM_) and crude digestible protein (D_CP_) of animal meal at three ages

Age	POM			MBM			Mean	SEM	*P*-Value		
	1	2	3	1	2	3			Age	Meal	Age x Meal
IDC_DM_, %											
1–8	60.27	62.13	63.97	54.85	56.12	51.84	58.19^B^	0.08	0.001	0.001	0.212
14–22	67.12	68.03	65.46	57.00	66.66	60.57	64.14^A^				
28–36	65.22	62.94	65.56	68.36	64.04	62.21	64.72^A^				
Means	64.21^a^	64.37^a^	65.00^a^	60.07^bc^	62.28^b^	58.21^c^					
IDC_CP_, %											
1–8	72.32	70.15	76.66	75.71	76.04	80.52	75.23^A^	0.214	0.001	0.003	0.912
14–22	69.20	71.86	72.34	76.07	77.60	79.66	74.45^A^				
28–36	68.20	70.10	68.77	74.54	76.66	74.54	72.13^B^				
Means	69.90^c^	70.70^c^	72.59^bc^	75.44^ab^	76.77^ab^	78.24^a^					
D_DM_, g/kg DMFeed											
1–8	543.77	562.20	576.30	495.97	501.34	465.21	524.13^B^	1.254	0.001	0.002	0.132
14–22	606.97	612.57	590.38	514.96	601.53	548.04	579.07 ^A^				
28–36	590.57	566.90	591.62	616.43	575.72	558.09	583.22^A^				
Means	580.4^a^	580.6^a^	586.1^a^	542.5^bc^	559.5^b^	523.8^c^					
D_CP_, g/kg CP											
1–8	205.51	221.42	221.41	225.80	220.75	240.67	222.59^A^	9.95	0.001	0.001	0.521
14–22	190.37	205.63	184.71	217.76	230.03	241.10	211.60^B^				
28–36	217.57	195.01	193.72	210.18	204.99	219.56	206.84^B^				
Means	204.48^bc^	207.35^bc^	199.94^c^	217.92^ab^	218.59^ab^	233.78^a^					

Different lowercase letters in the row and uppercase letters in the column. differ by the Tukey test at 5% probability; POM−poultry offal meal

MBM− meat and bone meal; P−value: probability; CV: coefficient of variation; IxF: probability of age factor interaction and flour factor

Digestible DM and CP were significant for the age and meal factors (*P* < 0.01), but there was no interaction between them. Digestible DM was higher in the treatments with POM, with the greatest digestibility obtained at 36 days of age. The highest D_CP_ was found in the MBM-containing diets, and the highest digestibility was observed in the initial stage of life.

Correlation analysis (Table 5) revealed that P was negatively correlated with EE, but had a highly positive correlation with Ca. The GE, in turn, was negatively correlated with the Ca levels.

**Table 5:** Pearson correlation analysis between energetic values and chemical components of poultry offal meal ^1^

Item	CP	EE	MM	Ca	*P*	GE	AME_*n*_1	AME_*n*_2
EE	-0.194							
P*	0.713							
MM	0.398	-0.793						
P*	0.435	0.060						
Ca	-0.307	-0.776	0.341					
P*	0.553	0.070	0.509					
P	-0.162	-0.888	0.504	0.979				
P*	0.759	0.018	0.308	0.001				
GE	0.597	0.380	0.101	-0.878	-0.763			
P*	0.210	0.458	0.848	0.021	0.078			
AME_n_^1^	0.682	0.954	0.582	-0.932	-0.944	0.689		
P*	0.177	0.007	0.226	0.003	0.002	0.130		
AME_n_^2^	0.742	0.843	0.522	-0.840	-0.557	0.877	-0.255	
P*	0.091	0.032	0.288	0.049	0.287	0.022	0.625	
AME_n_^3^	0.698	0.816	0.341	-0.717	-0.560	0.963	-0.468	0.974
P*	0.123	0.047	0.509	0.109	0.248	0.002	0.349	0.001

^1^−Values adjusted to 92% DM; P * − Probability significant when *P*<0.05; CP−Crude protein; EE− Ethereal extract; GE−Gross energy; MM−mineral matter; Ca−Calcium; P−Phosphorus; AMEn−Nitrogen corrected apparent metabolizable energy

Among the energy values of the three evaluated phases and the chemical components of POM, AME_n_ 1 was found to be correlated positively with the EE contents and negatively with Ca and P. Therefore, higher levels of these minerals decrease ME value. A positive correlation was seen between AME_n_ 2, EE, and GE contents, but AME_n_ 2 was negatively correlated with the Ca level. Lastly, AME_n_ 3 was correlated positively with EE and GE and negatively with Ca, although no significance was detected for the last item.

The chemical components analysis in the MBM (Table 6) revealed that the Ca level in them is negatively correlated with CP. The P had a high positive correlation with MM, while the opposite was found for GE, which was highly negatively correlated with MM and P.

**Table 6:** Pearson correlation analysis between energetic values and the chemical components of meat and bone meal^1^

Variable	CP	EE	MM	Ca	*P*	GE	AME_*n*_1	AME_*n*_2
EE	-0.775							
P*	0.070							
MM	-0.643	0.092						
P*	0.168	0.862						
Ca	-0.960	0.693	0.775					
P*	0.002	0.127	0.070					
P	-0.708	0.167	0.988	0.827				
P*	0.116	0.751	0.001	0.042				
GE	0.519	0.084	-0.975	-0.660	-0.968			
P*	0.292	0.874	0.001	0.154	0.002			
AME_n_^1^	0.920	0.560	-0.869	-0.925	-0.911	0.778		
P*	0.009	0.248	0.025	0.001	0.012	0.068		
AME_n_^2^	0.656	0.468	-0.829	-0.613	-0.793	0.820	-0.471	
P*	0.168	0.350	0.041	0.145	0.060	0.021	0.346	
AME_n_^3^	0.612	0.493	-0.874	-0.654	-0.826	0.841	-0.773	0.923
P*	0.199	0.362	0.021	0.159	0.022	0.030	0.071	0.009

^1^−Values adjusted to 92% DM; P * − Probability significant when *P*<0.05; CP−Crude protein; EE− Ethereal extract; GE−Gross energy; MM−mineral matter; Ca−Calcium; P−Phosphorus; AMEn−Nitrogen corrected apparent metabolizable energy

CP was highly correlated with AME_n_ 1, whereas the correlation between AME_n_ 1 and MM, Ca, and P was negative. In phase two, AME_n_ was correlated negatively with MM and positively with GE. Finally, AME_n_ 3 correlated negatively with MM and P but positively with GE.

Based on chemical composition and AME_n_ values obtained in the experiment, nine AME_n_ prediction equations were adjusted separately per phase in addition an equation was fitted with the composition values of both meals, also per phase. Six equations were also developed for all phases with the age variable in the models (Table 7).

**Table 7:** Nitrogen corrected apparent metabolizable energy (AME_n_) prediction equations of the animal meals according to the chemical composition in the different phases^1^

Equations AME_*n*_	Intercept	CP	EE	MM	Ca	*P*	GE	Age	*R* ^2^
Pre-initial phase 1–8									
POM	3455	-----	- 12.83	----	-----	48.15	----	----	96
MBM	4191	----	50.64	----	- 201.0	----	----	----	97
POM/MBM	3776	----	- 101.56	- 31.79	----	----	0.2708	----	94
Initial phase 14–22									
POM	-26,316	-----	- 111	----	-----	-----	5.959	----	98
MBM	2642	----	- 140.9	----	107.2	----	----	----	97
POM/MBM	-5581	----	----	50.8	- 162.7	280.4	1.599	----	95
Growth phase 28–36									
POM	9131	----	- 328.4	----	- 231.4	----	----	----	97
MBM	2328	----	- 126.0	----	135.5	----	----	----	96
POM/MBM	-9996	----	----	118.6	- 95.7	----	2.383	----	95
All phases									
POM	-35,531	----	----	----	76.9	----	7.32	10.47	84
POM	6802	----	- 213.3	----	- 127.3	----	----	10.47	92
MBM	2733	----	- 60.3	----	----	----	----	20.28	88
MBM	2789	----	- 72	----	14	----	----	20.28	91
POM/MBM	-546	56.46	----	----	----	----	----	17.55	87
POM/MBM	-4523	----	112.3	----	- 201.2	536.9	1.086	13.18	88

^1^−Values adjusted to 92% MS; PB−Crude protein; EE− Ethereal extract; GE−Gross energy; MM−mineral matter; Ca−Calcium; P−Phosphorus; AMEn−Nitrogen corrected apparent metabolizable energy; R2−Coefficient of determination

_POM−poultry offal meal; MBM−mea and bones meal_

The most representative variables for the AME_n_ meals prediction were EE, Ca, and GE. The equations adjusted with the AME_n_ data of all phases, those which obtained the highest determination coefficients were AME_n_ = 6,802 ‒ 213.3 (EE) ‒ 127.3 (Ca) + 10.47 (A) (R^2^ = 0.92), for POM; and AME_n_ = 2,789 ‒ 72 (EE) + 14 (Ca) + 20.28 (A) (R^2^ = 0.91), for MBM.

## Discussion

In this study, it was possible to observe the effects of age and chemical composition on metabolizable energy, metabolizable coefficients and the ileal digestibility of the broilers. The mathematical equations proposed can be used to predict the energy values of animal-derived meals in practical broilers’ diets.

### Chemical composition

The moisture content of all evaluated meals was below 8%. Residual moisture after processing typically ranges between 4% and 6%, with a maximum desirable level of 8–9%. Exceeding these limits can lead to undesirable microbial growth, product deterioration, and susceptibility to fat rancidity. Conversely, very low moisture levels may indicate overcooking (Bellaver and Zanotto 2004; Sales et al. 2013).

The crude protein content in meat and bone meals varies between 46.8% and 82.9%, while in poultry viscera meals, it ranges from 54.3 to 71.8% (Bhaskar; Pyne; Ray, 2014; Djissou et al. 2018). Sriperm et al. (2011) declared that the CP content in POM and MBM — commonly calculated as their nitrogen content multiplied by a standard conversion factor (6.25) is overestimated since each feedstuff has a specific coefficient. The coefficients determined for POM and MBM were 5.45 and 5.37, respectively.

Rostagno et al. (2024) classify MBM into 5 groups according to their CP content: 38, 43, 48, 55 and 58%. The crude protein of a batch of meat meals is inversely proportional to the amount of ash (mineral matter). High moisture and fat levels also cause a reduction in protein content (Souza 2020). According to this classification, the MBM evaluated here falls into group 43% and is within the levels (40 to 45% CP) ensured by the commercial brands that manufacture them.

The EE MBMs contents in the current study agree with those found in the literature, which range from 9.6% (Troni et al. 2016) to 15.49% (Tucci et al. 2003). The EE is a variable related to the quality of meals, since elevated EE contents may reduce storage time and make the meal susceptible to rancidification (Oliveira et al. 2009). The amount of EE is also related to the oil-extraction method using during the manufacturing process (Toldrá et al. 2016).

The main factor of variation in the production of MBMs is the percentage of bones in the mixture. A higher concentration, therefore, will lead to a lower percentage of CP and GE and consequently higher mineral matter contents (Troni et al. 2016). Dale (1997), Wang and Parson (1998), and Shirley and Parsons (2001) observed that CP and GE levels decline as the MM concentration is increased, while Ca and P contents increase.

In addition to the proportion of raw material, crude protein and amino acids concentrations in animal-derived meals are highly influenced by processing parameters such as temperature, time, and pressure, which vary significantly between rendering systems (Iskakov et al. 2021). The lysine and methionine levels evaluated for POM were higher than the values provided by the NRC (1994), which are 3.10% and 0.99%, respectively. For example, excessive thermal exposure during cooking can lead to Maillard reactions that decrease amino acid availability, especially lysine, while inadequate processing may not adequately denature proteins, which can reduce digestibility (Moughan et al. 2018).

Shirley and Parsons (2000) reported that increased pressure during meat and bone meal processing significantly reduces the digestibility of protein and amino acids, especially lysine and cystine. Furthermore, the methods used for fat extraction, such as mechanical pressing versus chemical solvents, directly affect ether extract levels, which are strongly correlated with energy content. As the processing of poultry offal and meat and bone meal is not standardized across suppliers, these factors contribute substantially to the variability observed in the nutrient composition and energy values of these meals. Therefore, a better understanding and control of rendering conditions are crucial to ensure the consistency and predictability of these ingredients in feed formulation (Pérez-Calvo et al. 2010).

### Nutrient metabolisability

The results of this study are aligned with those obtained by Schneiders et al. (2021), who reported that energy values rose as the birds aged, increasing their metabolisability coefficients. The authors attributed this finding to the development of the digestive tract, whose ability to utilize nutrients and energy from the feed improves as the animal ages.

The variations in energy levels observed in this study can be explained by the variability in the raw materials of the meals, the age and sex of the birds used in the trials, the methodology employed in the experiment and chemical analyses, and the percentage of substitution of the test ingredient (Nascif et al. 2004; Nascimento et al. 2005; Brumano et al. 2006).

Faria Filho et al. (2002) show that the ME values in MBM are usually underestimated when obtained using methodologies in which the inclusion level of this ingredient in the control diet ranges from 40 to 50%. This is possible because the elevated Ca and P levels provided by the high MBM inclusion compromised the utilization of other nutrients. Furthermore, the most adequate level of MBM inclusion in the control diet to determine energy values is 20% (Martosiswoyo and Jensen 1988).

This study found lower MC_GE_ than the values (53.65 to 61.96%) obtained by Eyng et al. (2010) in three POMs. According to those authors, this difference may be attributed to the fatty acid meal composition, and higher unsaturated: saturated fatty acid ratios increase metabolisability coefficients.

Sakomura et al. (2004) mentioned that the lower ME values and metabolisability coefficients observed in the first three weeks of a bird’s life can be explained by the low digestibility coefficients of EE in that period and the low lipase activity. During that period, birds’ digestive capacity is not fully developed, which limits their utilization of nutrients, mainly fat (Noy and Sklan 1997).

Although the ether extract content was considered in the energy prediction models, this study did not analyze the specific fatty acid composition of the meals. Given that different fatty acid profiles—particularly the ratio of unsaturated to saturated fatty acids—can significantly influence the metabolizability of dietary fats, future studies should include detailed fatty acid profiling of animal-derived meals better to understand their impact on energy utilization in broilers.

### Ileal digestibility coefficients

The birds’ ability to digest and absorb protein is influenced by age (Batal and Parsons 2002; Garcia et al. 2007; Ross et al. 2019). The improvement in IDC_DM_ seen with increasing age of birds in the present study, was a consequence of gastrointestinal development, thus providing better use of the nutrients in animal meals (Adedokun, 2014). As Barbosa et al. (2008) described, IDCDM reflects nutrient digestibility, and thus an increase in this coefficient indicates greater nutrient absorption from diet.

Birds receiving diets formulated with MBM had a higher protein intake and consumed more amino acids. As explained by Hughes and Choct (1999), the different protein dietary sources are homogeneous protein mixes that are digested at different rates, which leads to variations in the rate at which the intestine absorbs different amino acids. Rochell et al. (2013) worked with different animal meals and highlighted that MBM showed significant variations in amino acid digestibility according to the batch evaluated. Variations in digestibility are also related to meal processing. Increased pressure during the processing of MBM significantly reduced the digestibility of protein and of most amino acids, the highest reductions occurring in lysine and cystine (Shirley and Parsons 2000).

For DM and CP, there were differences between ileal digestibility and digestibility measured in the excreta, in the present experiment, with higher values observed in the ileum. Kadim et al. (2002) submitted that the ileal digestibility approach has advantages over the total collection method because the protein composition is not altered by the microbiota in the distal intestine part, and it prevents the combination of feces with nitrogen and urinary amino acids. In contrast, Hendriks et al. (2012) reported that the large intestine’s influence on protein digestibility is insignificant, and that little advantage exists over conventional excreta analysis. However, Saki et al. (2010) observed that the AME and AME_n_ values in the feces are more appropriate than those found in the ileum. By contrast, the estimate of protein digestibility in the ileum generates better results than in the birds’ excreta, with digestibility values from the total gastrointestinal tract generally lower than obtained at the ileal level.

### Correlation coefficients

The current results corroborate those obtained by Silva et al. (2010), who observed that AME_n_ in POM was positively correlated with CP and GE but negatively correlated with MM, Ca, and P. Given these results, higher mineral matter contents in MBM are expected to result in lower AME_n_ values. Moreover, considering that determining MM is a practical procedure, it can be applied as an instrument for estimating the chemical composition, since, as stated by Najafabadi et al. (2007), the MM content is a good chemical composition indicator of animal-derived meals.

### Prediction equations

The most representative variables for predicting the AMEn meals in this study were EE, Ca, and GE. Souza (2009) worked with animal-derived feedstuffs, one of which was POM, and concluded that the factors that contributed to best-fitting equations for predicting AMEn in broilers and roosters’ 10 to 17, 26 to 33, and 40 to 47-day-old phases were CP, EE, and MM.

To obtain a single prediction equation to estimate the AMEn values of protein feedstuffs commonly used in broiler diets, Oliveira et al. (2018) conducted a meta-analysis study using Brazilian articles that cataloged information on the AMEn values and the chemical composition of these ingredients. In their meta-analytic study, the best fitting equations to estimate the AMEn of poultry offal meal and meat and bone meal were AMEn = 6139 − 45.5 CP + 0.356 GE − 123.5 MM (R² = 0.8302) and AMEn = 2267 + 19.9 CP + 67.9 EE − 44.4 MM (R² = 0.9021), respectively. This study highlighted the usefulness of meta-analysis for developing efficient predictive equations. However, Mariano et al. (2020) proposed that using more advanced machine learning models, such as neural network committees, can overcome the limitations of traditional linear models, offering greater accuracy and reliability in energy prediction for poultry feed formulation.

The fit of equations in the present experiments, with the high coefficients of determination of 94 to 98%, might have been influenced by the number and variability of data and by the indirect-elimination (Backward) method, which was used to generate the equations and indicate the contribution of each variable within the multiple regression analysis and the equation that best represents the studied phenomenon. Afterward, it removes the variables that least contribute to obtaining the energy value until only one is left, with the best R^2^.

This study examined the chemical composition, energy values, and digestibility of animal-based meals, noting their impact on broiler gut microbiota, which is vital for nutrient digestion, immune response, and energy metabolism (Pan and Yu, 2013). Animal protein sources alter microbial communities and produce short-chain fatty acids, affecting feed efficiency and energy use (Apajalahti and Vienola 2016). Future research should explore how various animal-based meals influence gut microbiota to better understand nutrient utilization and enhance nutritional strategies for broilers. Feed ingredient costs are crucial in poultry production. While this study lacks an economic analysis, using poultry offal and meat and bone meals may reduce feed expenses compared to traditional sources like soybean meal and dicalcium phosphate. Including cost-benefit evaluations in future studies could enhance the practical application of energy prediction models.

## Conclusions

The nitrogen-corrected ME of poultry offal meal ranged from 3,189 to 3,342 kcal/kg. In the meat and bone meal, AME_n_ ranged from 2,292 to 2,345 kcal/kg. The equations fitted with the AME_n_ data of all phases which obtained the highest determination coefficients for POM and MBM were AME_n_ = 6,802 ‒ 213.3 (EE) ‒ 127.3 (Ca) + 10.47 (age) (R^2^ = 0.92) and AME_n_ = 2,789 ‒ 72 (EE) + 14 (Ca) + 20.28 (age) (R^2^ = 0.91), respectively.

This study developed robust, age-specific prediction models for estimating the AMEn of poultry offal meal and meat and bone meal based on their chemical composition. Given the variability of these by-products and age-related changes in broiler digestion, the models offer a practical alternative to in vivo trials. They allow rapid estimation of energy values for different batches, improving feed formulation accuracy. This contributes to better broiler performance and economic efficiency in poultry production systems.

## Data Availability

Data will be made available on reasonable request.
